# The occurrence of minor mastitis pathogens in dairy cows in Slovakia

**DOI:** 10.17221/14/2026-VETMED

**Published:** 2026-09-24

**Authors:** Kristina Tvarozkova, Barbora Gancarova, Ludovit Cernek, Lucia Macuhova, Michal Uhrincat, Martina Vrskova, Vladimir Tancin

**Affiliations:** ^1^Slovak University of Agriculture in Nitra, Nitra, Slovak Republic; ^2^VETWELL s.r.o., Lužianky, Slovak Republic; ^3^National Agricultural and Food Centre, Lužianky, Slovak Republic

**Keywords:** cows, mastitis, milk, minor pathogens

## Abstract

Udder health in the dairy industry is one of the main factors influencing the production of high-quality and safe dairy products. In dairy practice, the udder is exposed to the presence of microorganisms, which, after penetration through the teat canal into the tissue, cause the main health problem – mastitis. The aim of our study was to determine the prevalence of minor pathogens in the milk of dairy cows with suspected udder health problems under the conditions of Slovak dairy farms in 2017–2023. A total of 47 dairy farms were involved, and 2 236 udder quarter milk samples were collected from dairy cows. Minor pathogens accounted for 11.38% of the identified pathogens in milk samples, indicating their low incidence in dairy practice. In total, the most frequent minor pathogens were *Enterococcus* spp. (2.65%), *Serratia marcescens* (1.77%), and *Klebsiella pneumoniae* (1.41%), with differences in occurrence in the studied years. In conclusion, the occurrence of minor mastitis pathogens in milk samples indicates lower importance in causing mastitis problems, but their identification is also necessary for the application of preventive anti-mastitis programmes and mastitis treatment.

Udder health is a key factor in producing nutritionally healthy raw milk, high-quality and safe food in the dairy industry, and maintaining high milk yield and good cow welfare. Clinical and subclinical mastitis has long caused economic losses in the dairy industry due to declines in milk production, reduced suitability for human consumption, and increased investment in treatment and early culling of sick dairy cows (Lucken et al. 2021; Hanus et al. 2025). The most common causes of mastitis in dairy cows are coagulase-negative staphylococci (CNS) (Dufour et al. 2019; Tvarozkova et al. 2025). Mastitis caused by major pathogens is a serious problem, but mastitis caused by minor pathogens should not be overlooked (Zigo et al. 2022). For example, *Trueperella pyogenes* is a mastitis pathogen that tends to be associated with the environment, but it cannot be clearly classified as a contagious or environmental pathogen because its reservoirs and transmission routes are not yet known (Wente et al. 2024). Tvarozkova et al. (2025) recently published a report on monitoring the main pathogens in dairy cows in Slovakia. Referring to the last-mentioned article, this study aimed to continue monitoring the occurrence of minor pathogens in dairy cows with suspected mastitis under Slovak dairy practice.

## MATERIAL AND METHODS

### Sampling

Milk samples were collected by practising veterinarians from dairy cows suspected of mastitis on dairy farms based on the California Mastitis Test (CMT) or visible abnormalities in milk or high somatic cell count based on monthly milk recording data (Vyletelova Klimesova et al. 2013; Tvarozkova et al. 2025). In 2017, samples were obtained from 52 collections (47 farms), 32 collections in 2018 (29 farms), 31 collections in 2019 (29 farms), 44 collections in 2020 (41 farms), 40 collections in 2021 (35 farms), 33 collections in 2022 (31 farms), and 38 collections in 2023 (35 farms). One collection represented samples from several dairy cows from the farm. A total of 2 236 udder quarter milk samples were collected from dairy cows and examined (NMC 2017).

### Bacterial culture

Milk samples (10 μl) were cultured aerobically on blood agar plates and MacConkey agar (MkB Test a.s., Rosina, Slovak Republic) according to the methodology described in “The Laboratory and Field Handbook on Bovine Mastitis” by the National Mastitis Council (NMC 2017). Inoculated plates were incubated at 37 °C and checked after 24 and 48 hours. The bacteria from the grown colonies were initially identified by their morphology, stained and evaluated according to Gram staining, tested for haemolysis, catalase, aesculin hydrolysis, cell morphology, and cytochrome oxidase C (Bactident Oxidase; Merck KGaA, Darmstadt, Germany). The confirmatory test for presumptive *Staphylococcus* (*S.*) *aureus* was verified using the clumping factor test (DiaMondiaL Staph Plus Kit, Germany).

To identify *Streptococcus* (*Str.*) *uberis* or *Entero-coccus* spp., aesculin-positive streptococci were further cultured on modified Rambach agar (Watts et al. 1993). Lancefield serotyping (DiaMondiaL Strept Kit, Germany) was used to identify aesculin-negative streptococci. Group B streptococci were referred to as *Str. agalactiae,* and those from group C as *Str. dysgalactiae*. Catalase-positive and Gram-positive small colonies were identified as coryneform bacteria. Gram-positive, catalase-positive large colonies on blood agar were identified as *Bacillus* spp. The grown colonies were identified using MALDI-TOF MS (Bruker Daltonics, Bremen, Germany) (Tvarozkova et al. 2021). Besides contagious pathogens, other pathogens were classified as positive if at least five colony-forming units (CFU) were present. A sample was classified as contaminated and subsequently discarded from evaluation if three or more different colony types were detected. The results were processed using Microsoft Excel (Microsoft, Redmond, WA, USA).

## RESULTS AND DISCUSSION

In the same data set, as mentioned in the aim of our study, there were 2 486 isolates identified from 2 236 samples during the years of study, where up to 95.26% were found bacteriologically positive (Tvarozkova et al. 2025) and where the major pathogens [CNS, *Escherichia* (*E.*) *coli, Str. uberis,* *S. aureus,* and *Str. agalactiae*] represented 88.62% of the identified isolates. In the present study, the minor pathogens accounted for 11.38% of the identified pathogens. In the overall assessment of the occurrence of individual minor pathogens during all monitored years, we found the highest occurrence of *Enterococcus* spp. (2.65%) in the range of 0.86% (2019) to 6.30% (2017) (Table 1). In their study conducted in Slovakia, Zigo et al. (2022) found a higher prevalence of *Enterococcus* spp. (6.10%). In Germany, Buthe et al. (2025) and in Australia, Joy et al. (2023) also reported a slightly higher incidence of *Enterococcus* spp. (5.63% and 4.60%, respectively) in their studies compared to the results of our study. In Italy, Ulloa et al. (2025) also reported a slightly higher prevalence of *Enterococcus* spp. (3.41%). The second most common minor pathogen in our study was* Serratia marcescens *(1.77%), ranging from 0.00% (2017) to 3.73% (2022) (Table 1). In Germany, Katthofer et al. (2024) detected Gram-negative pathogens (coliform bacteria and *Serratia marcescens*) in 0.40% of samples. A lower incidence of *Serratia marcescens* (0.31%) was reported by Buthe et al. (2025) in Germany. The third most common minor pathogen in our study was* Klebsiella pneumoniae *(1.41%), ranging from 0.00% (2018) to 4.18% (2023) (Table 1). Holko et al. (2019) also observed a lower incidence of *Klebsiella pneumoniae* (<1.00%) in their study conducted under Slovak breeding conditions. In Bangladesh, Emon et al. (2024) recorded a high prevalence of *Klebsiella pneumoniae *(66.82%)*, *with this pathogen being the second most common cause of mastitis in their study. In Germany, Buthe et al. (2025) observed a similar prevalence of *Klebsiella pneumoniae *(1.51%) as we recorded in our study. The occurrence of *Str. dysgalactiae *was 1.01%, within the range of 0.00% (2021) to 2.01% (2019) (Table 1), similar to a previous study conducted under Slovak breeding conditions by Holko et al. (2019), which reported an incidence of *Str. dysgalactiae *below 1.00%. In Kosovo, Mazreku et al. (2025) detected a higher *Str. dysgalactiae *occurrence of 3.20% in their study. *Pseudomonas aeruginosa *occurrence was 1.01% in the range of 0.00% (2018) to 3.15% (2019). In Kenya, Mbindyo et al. (2020) reported a higher prevalence of *Pseudomonas aeruginosa* (5.1%) compared to our results. Compared with other data, our results suggest that the year of the study is an important factor.

**Table 1:** The occurrence of minor pathogens during the monitoring period (2017–2023) (*n* – number of isolates, % of occurrence from all identified pathogens)

Pathogens	2017			2018			2019			2020			2021			2022			2023	
	*n*	%		*n*	%		*n*	%		*n*	%		*n*	%		*n*	%		*n*	%
*Serratia marcescens*	0	0		1	0.44		2	0.57		2	0.64		18	3.32		12	3.73		9	2.35
*Klebsiella pneumoniae*	4	1.15		0	0		1	0.29		1	0.32		8	1.48		5	1.55		16	4.18
*Pasteurella* spp.	2	0.57		0	0		1	0.29		2	0.64		6	1.11		0	0		4	1.04
*Enterococcus* spp.	22	6.30		5	2.20		3	0.86		6	1.91		8	1.48		5	1.55		17	4.44
*Streptococcus dysgalactiae*	5	1.43		4	1.76		7	2.01		4	1.27		0	0		3	0.93		2	0.52
*Corynebacterium bovis*	2	0.57		0	0		0	0		2	0.64		0	0		7	2.17		9	2.35
*Proteus* spp.	0	0		0	0		0	0		6	1.91		0	0		0	0		0	0
*Trueperella pyogenes*	0	0		0	0		0	0		2	0.64		3	0.55		0	0		1	0.26
*Pseudomonas aeruginosa*	4	1.15		0	0		11	3.15		3	0.96		2	0.37		2	0.62		3	0.78
*Corynebacterium* spp.	0	0		0	0		0	0		0	0		0	0		2	0.62		5	1.31
Others	2	0.57		13	5.73		2	0.57		3	0.96		3	0.55		6	1.86		2	0.52

Others: *Micrococcus sedentarius*, *Serratia rubidaea*, viridans streptococci*, Empedobacter brevis*, *Pantoea agglomerans*, *Yersinia enterocolitica*, *Pasteurella aerogenes*, Gram-negative rods

The occurrence of other pathogens such as *Pasteurella* spp., *Corynebacterium bovis, Proteus* spp., *Trueperella pyogenes*, and *Corynebacterium* spp. was less than 1% during the whole study period (Table 1). We observed *Pasteurella *spp. in 0.60% of bacteriologically positive samples. In Slovakia, Holko et al. (2019) detected *Pasteurella multocida *in <1.00%. The occurrence of *Corynebacterium *spp. was very low in our study (0.28%) compared with studies by Dalanezi et al. (2020) in Brazil and Buthe et al. (2025) in Germany, who observed a higher occurrence of *Corynebacterium *spp. (9.50% and 15.70%, respectively) in their study. In Canada, Acharya et al. (2021) reported the occurrence of *Corynebacterium* spp. at 2.96% in their study. In Italy, Ulloa et al. (2025) also observed a higher prevalence of *Corynebacterium* spp. (6.1%). In the Czech Republic, Bzdil (2017) sampled only cows with symptoms of mastitis and detected *Corynebacterium bovis* in 1.65% of samples, more than double the incidence observed in our study. Similarly, Holko et al. (2019) found a low occurrence of *Corynebacterium bovis *(<1.00%) in Slovakia. In Germany, Muller et al. (2023) observed a higher prevalence of *Corynebacterium bovis *(13.64%) compared to our results. *Proteus* spp. accounted for only 0.24% of all isolates during the monitored years. In Slovakia, Holko et al. (2019) also observed a low occurrence of *Proteus* spp. (<1.00%) in their study. Similarly, Ulloa et al. (2025) observed a low incidence of *Proteus* spp. (0.40%) in Italy. Mazreku et al. (2025) recorded a higher occurrence of *Proteus* spp. (2.10%) in Kosovo. Farkasova et al. (2024) reported a higher incidence of *Proteus* spp. (7.60%) in their study conducted in Slovakia. A higher prevalence of *Proteus* spp. (11.10%) was also reported by Hisira et al. (2020) in Slovakia. In our study, we found a low prevalence of *Trueperella pyogenes *(0.24%). In Italy, Ulloa et al. (2025) found a similarly low occurrence of *Trueperella pyogenes *(0.63%). In Germany, Katthofer et al. (2024) observed *Trueperella pyogenes*, *Lactococcus garvieae, *and* Aerococcus viridans* in 0.5% of the samples. In Germany, Buthe et al. (2025) reported a prevalence of *Trueperella pyogenes *in 0.91% of positive samples.

Our data indicate that even minor mastitis pathogens could be a serious problem on dairy farms. In some years, especially *Serratia marcescens, Enterococcus* spp., *Corynebacterium bovis*, and *Klebsiella pneumoniae* reached percentages that deserve increased attention from farmers and scientists. Therefore, identifying them is also necessary to apply preventive anti-mastitis programmes focused on farm hygiene in dairy practice, as well as to build theoretical knowledge of pathogen presence on Slovakian dairy farms.
